# Caregiver-Youth Communication Patterns and Sexual and Reproductive Health Among American Indian Youth

**DOI:** 10.1007/s10903-024-01616-3

**Published:** 2024-07-12

**Authors:** Jeffrey Thiele, Olivia Williamson, Olivia Ceavers, Paula FireMoon, Olivia Johnson, Elizabeth Rink, Michael Anastario

**Affiliations:** 1 Department of Health & Human Development, Montana State University, 250 Reid Hall, Bozeman, MT 59717 USA; 2 Robert Stempel College of Public Health & Social Work, Florida International University, Miami, FL USA; 3 Fort Peck Community College, Poplar, MT USA; 4 Department of Health Sciences, Northern Arizona University, Flagstaff, AZ USA

**Keywords:** American Indian, Youth, Condom use, Caregiver-youth communication, Sexual health communication

## Abstract

Improving communication between American Indian caregivers and their youth has been suggested as an Indigenous-forward strategy to help alleviate the sexual and reproductive health (SRH) disparities faced by American Indian youth as a result of the legacy of colonial violence against American Indian communities. Studies with non-American Indian and American Indian populations suggest that effective communication about SRH between parents and youth plays a role in reducing sexual risk behaviors among youth. There is limited research that examines youth sexual risk behaviors in relation to communication patterns separately assessed in caregivers and youth. The current study aimed to examine the association between caregiver-youth communication patterns and engagement in sex, age at sexual debut, and condom use among American Indian youth in the United States. The study draws on baseline caregiver and youth data collected from Nen ŨnkUmbi/EdaHiYedo, a stepped wedge design trial with American Indian youth living on the Fort Peck Reservation in Montana. 113 caregiver responses were matched to 145 youth for the current study. Caregiver-youth communication patterns were examined in relation to youth engagement in sex, age at sexual debut, and number of protected acts of vaginal and/or anal sex. Multivariable models were used to adjust for confounders and to examine relationships between caregiver-youth communication and youth sexual risk outcomes. An increase in overall level of self-reported youth communication with caregivers about sexual and reproductive health topics was significantly associated with a greater likelihood of youth ever having engaged in sex. A significant interaction effect between youth communication and convergence with caregiver response was observed for the number of protected acts of vaginal and/or anal sex, where caregiver communication (regardless of self-reported youth communication with caregivers) was associated with a greater number of protected sex acts. This study fills a gap in the extant literature by reporting on relationships between communication about SRH, assessed separately in caregivers and youth, and youth sexual risk behaviors. Findings emphasize the importance of involving American Indian caregivers in SRH interventions to improve SRH outcomes among American Indian youth, and inform future experimental research that will evaluate how changes in caregiver communication potentially impact youth SRH.

## Background

The legacy of colonial violence against American Indian (AI) populations has undermined Indigenous knowledge and practice surrounding sexual and reproductive health (SRH) [1]. For example, Christian evangelization eroded the connection between spirituality and SRH that was passed down from AI Elders to their youth prior to colonization, converting a once prevalent Indigenous cultural practice into a social taboo [1]. Improving family cohesion and communication between AI caregivers and their youth among northern plains AI populations has been suggested as a culturally centered, Indigenous-forward strategy to help alleviate the SRH disparities faced by AI youth as a result of the continued legacy of colonial violence against AI communities [1]. In the context of this study, a caregiver can be a parent, grandparent, aunt, uncle, sister, brother, cousin, friend, or other trusted member of the community who is caring for one or many AI youth. Improving caregiver-youth communication offers a means of restoring cultural and spiritual practices that emphasized the connection between AI youth and their caregivers [1], where adapting this communication to address more relevant, present-day needs related to condom use, sexually transmitted infections (STIs), and gender/sexuality is imperative to contemporary native survivance. Studies with AI and non-AI youth suggest that caregiver-youth communication regarding SRH issues increases following sexual debut of youth, and that caregiver-youth communication can play a role in reducing sexual risk behaviors among sexually active youth. However, there is limited extant research concerning the ways in which communication about SRH reported separately by caregivers and youth are associated with the SRH of AI youth.

Studies that have explored the effects of parent-youth communication on SRH outcomes among AI youth have generally focused on sexual activity, early sexual debut, and condom use. Among AI youth in grades 6–12 who participated in one cross-sectional study overseen by the Great Lakes Inter-Tribal Council, youth were more likely to report never having had sex when reporting fewer sexual-risk conversations with parents [2]. This finding is evident in other studies where more sexual activity among youth was associated with greater communication with parents about SRH topics [3, 4]. In one longitudinal study of 456 12–16-year-old self-identified AI youth, delayed sexual debut was associated with increased parental communication about sex [5]. These findings suggest that parent-youth communication may play a key role in preventing sexual activity, and that parent-youth communication about SRH topics increases following youth sexual debut. These findings are also consistent with studies of non-AI youth where increased parental communication has been associated with youth sexual initiation [6, 7], which may reflect greater parental anticipation of youth behavior and responsive conversation to the everyday activities of youth.

Studies focused on condom use among AI youth have generally reported a positive association between communication with a parent regarding sex and condom use, but constructs and measures have varied across studies. Chewning et al. reported a positive association between parental communication regarding SRH and condom use in AI youth [2]. In a separate study of 543 AI youth ages 11–19, parental communication about sex was significantly associated with an increased likelihood of male youth reporting an intention to use a condom at next sexual encounter [8]. In another study of AI male youth in Minnesota that examined partner communication (as opposed to parental communication), consistent partner communication concerning STI prevention was associated with self-reported condom use at last sexual encounter [9]. These findings are consistent with findings from studies involving non-AI youth, where positive relationships between parental communication and condom use have been observed for sexually active youth [10–12]. While the direction of the association between parent-youth communication and condom use is consistent, outcomes and outcome metrics have varied across studies.

In randomized controlled trials (RCTs), limited data have been collected concerning parent-youth communication among AI youth. Among non-AI youth, results from RCTs show that parent-youth communication has led to improved condom use skills as well as more youth condom use among those sexually active [13, 14]. Parent-based SRH interventions with non-AI youth have also led to increased parent-youth communication and youth condom use behavior [12]. In one RCT that evaluated Respecting the Circle of Life on AI parent-youth communication, trusted adults reported greater levels of SRH communication and spoke more frequently with youth about how to obtain condoms, birth control, and effectively protect against human immunodeficiency virus, as compared to their control counterparts [15].

Taken together, there is evidence that parental communication regarding SRH is associated with decreased sexual risk behavior among AI youth. However, there is a gap in the literature concerning the ways in which communication about SRH reported separately by parents and youth relate to SRH outcomes among AI youth. The purpose of this current study is to examine caregiver-youth communication patterns (based on self-report of the caregiver matched to the youth) in relation to SRH outcomes among northern plains AI youth. Results have direct implications for developing culturally appropriate interventions that improve SRH outcomes among AI youth while focusing on Indigenous values that help mitigate the deleterious effects left by the legacy of colonial violence against northern plains AI communities in the US.

## Methods

### Setting and Study Design

The study took place on the Fort Peck Indian Reservation (Fort Peck). Approximately 8,000 enrolled tribal members, most from the Assiniboine and Sioux Nations, live on the 2.1-million-acre reservation [16]. The study took place across five schools distributed between five separate communities, including Frazer, Wolf Point, Poplar, Brockton, and Culbertson. All communities but Culbertson are located on the reservation, where Culbertson is an eastern border town comprised of both Fort Peck and neighboring Fort Kipp youth and caregivers.

Data from this study are derived from baseline observations collected  as part of Nen ŨnkUmbi/EdaHiYedo (NE-“We are Here Now”, R01MD012761), a stepped-wedge design (SWD) trial to improve sexual and reproductive health outcomes among AI youth. In NE, all five school clusters received a baseline observation, then each school was randomized into the intervention at subsequent time points until all school clusters had received the intervention. A second baseline observation was taken prior to the start of the intervention for the second, third, fourth, and fifth clusters. This SWD study aligned with Fort Peck community values, which mandated that all youth in the study receive the intervention [17]. The NE protocol is described in more detail elsewhere [17]. The data presented in this manuscript are from baseline data collection on pre-intervention observations of caregivers and youth. Youth and caregivers completed separate questionnaires at baseline that addressed the following topics: What qualities are important in choosing close friends; What qualities to look for in a boyfriend, girlfriend, or life partner; How boys’ bodies change physically as they grow up; How girls’ bodies change physically as they grow up; How women get pregnant and have babies; Symptoms of sexually transmitted infections (STIs); Reasons why people like to have sex; How well birth control can prevent pregnancy; How to choose a method of birth control; How to say no if someone wants to have sex and you don’t want to; How well condoms can prevent STIs; Menstruation (having menstrual periods); The importance of not pressuring other people to have sex; How you will make decisions about whether or not to have sex; Consequences of getting pregnant/getting someone pregnant; How to use a condom; Reasons why you should not have sex; Masturbation; What it feels like to have sex; Homosexuality/people being gay; Wet dreams; What to do if a partner doesn’t want to use a condom; How people can prevent getting STIs; and How you will know if you are in love [18]. First and second baseline observations were collected during March 2019 for all clusters (first baseline), and second baseline observations were collected during January 2021 (second cluster), September 2021 (third and fourth clusters), and November 2022 (fifth cluster). Based on recommendations from the Fort Peck community advisory board and participating school administrators, passive and active consent was received for this study from caregivers and youth. Caregivers were informed of the study and were given an opportunity to opt their youth out of the study (passive). Caregivers themselves were given the opportunity to participate in the caregiver portion of the study (active). The youth also gave verbal consent before they took the baseline survey (active).

### Participants

To be eligible for inclusion in NE, youth were: (1) 14–18 years old; (2) a registered member of a federally recognized tribe or an associate tribal member; and (3) a resident of Fort Peck with a caregiver. Exclusion criteria included: (1) not meeting the aforementioned inclusion criteria; and (2) having a medically identified physical or cognitive impairment that would impede their understanding of and participation in *Native Stand, Native Voices* and the cultural mentoring program of NE. Eligibility of caregivers was predicated on youth eligibility.

At baseline, 677 youth were sampled. The research team did not encounter difficulties in recruiting youth for this study. According to school roster counts at the time of sampling, the youth participation rate was 85.9% [19]. Of the 677 youth, 21.4% had a caregiver that completed a questionnaire. Across all schools, 132 caregivers completed first and/or second baseline questionnaires, of which 80.3% had youth who completed a first and/or second baseline questionnaire. An attrition analysis (not reported here) revealed that the 26 caregivers who were sampled without corresponding youth observations were more frequently female and engaged in less communication than caregivers matched to youth. The final analytic sample presented here consists of 113 caregivers matched to 145 youth at baseline, with 28.3% of caregivers having ≥ 2 youth in the final sample. Assuming a similar composition of caregivers with ≥ 2 youth in the total initial youth sample (n = 677), an estimated total of 485 caregiver observations were possible at baseline. As such, the estimated caregiver participation rate for this baseline survey was 23.3%. Estimated caregiver participation rates across all cited studies from the meta-analysis by Widman et al. ranged from 8.6 to 84%, with an average of 50.1% caregiver participation [12]. The current study’s caregiver participation rate reflects a common difficulty for researchers, where many experience difficulty recruiting caregivers for programs and interventions [18].

### Data Collection

Surveys were administered by members of the research team on password-protected iPads using the REDCap electronics data capture tools [20]. Youth completed a questionnaire during a 45-min classroom period, which took approximately 30–45 min to complete. Caregivers completed a brief questionnaire in their home or at one of the two Fort Peck Community College campuses. Prior to taking the survey, a member of the research team explained survey instructions, gave an overview of the survey content, and described the purpose of the study to the survey participants. Youth received a $10 iTunes gift card and caregivers received $30 cash as an incentive for completing the survey. Human Subjects approval was obtained from the Fort Peck Institutional Review Board (IRB) and the Montana State University IRB (Protocol #: 2018-49-ER100318-FCR).

### Measures

#### Demographics

For youth demographics, age, gender (male, female, or neither male nor female), school site, and grade level (8th–12th) were measured. For youth gender, cases of neither male nor female were recoded to missing.

Caregiver demographics were measured with age, gender (male or female), school site for the youth in the survey, and caregivers’ highest level of education completed.

#### Youth and Caregiver Communication About Sexual and Reproductive Health

On separate questionnaires, youth and caregivers were asked 24 questions regarding whether they communicated with caregivers or youth (respectively) about a series of SRH topics. Questions were adapted from the work of Shuster et al. [18]. The topics concerned relationships, sexuality, and sexual and reproductive health, with response sets including “Yes”, “No”, and “I don’t know”. Each communication item was recoded as a dichotomous communication variable (0 = “No”, 0 = “I don’t know”, 1 = “Yes”). A cumulative communication score ranging from 0 to 24 was generated for each individual by summing each of the 24 individual communication variables.

Next, a separate variable was created to represent convergence between youth and caregiver communication responses for each of the 24 communication items. Youth responses were compared to matched caregiver responses for each question asked (0 = divergent response, 1 = convergent response). A cumulative convergence score ranging from 0 to 24 for each individual was generated by summing each of the individual convergence variables.

#### Youth Sexual Behavior

To measure whether youth had ever engaged in sex, youth were asked: “Have you ever had sexual intercourse?”, and their response (“Yes” or “No”) was coded as a dichotomous variable (0 = never engaged in sex, 1 = engaged in sex).

Youth age at sexual debut was measured by asking youth: “How old were you when you had sexual intercourse for the first time?” Scores ranged from 11 to 17 among youth who were sexually active.

The number of protected acts of anal and/or vaginal sex were measured by asking the youth the question: “Of the times you had sex over the past month, how many times was a condom used?” Youth provided a numerical response to the question. Sexually inactive youth were included as having 0 protected acts of anal and/or vaginal sex, and the count of protected acts was included for youth who were sexually active in the month preceding the survey (ranging from 0 to 6 acts).

### Analysis

Univariate analyses included examining frequencies and measures of central tendency and dispersion. Bivariate analyses were conducted to test for systematic relationships between demographic variables and SRH outcomes among youth. Pearson’s chi-square test was used to test for relationships between categorical variables, student’s t-test was used to test for differences in means between two groups, and one-way analysis of variance was used to test for difference in means across multiple groups. Prior to developing multivariable models, multicollinearity among the independent variables was examined with the Variance Inflation Factor.

Three sets of multivariable models were developed to evaluate the association between communication variables and the dependent variables of interest. All multivariable models were adjusted for the potentially confounding effects of youth age, youth gender, and the highest level of caregiver education completed, and standard errors were adjusted for potential clustering by study site/school. Logistic regression was used to test for associations between whether youth had ever engaged in sex and caregiver-youth communication. Ordinary least squares regression was used to test for associations between age at sexual debut and caregiver-youth communication. Negative binomial regression was used to test for associations between the number of protected acts of vaginal and/or anal sex and caregiver-youth communication, with the logarithm of the expected count calculated for communication variables. Negative binomial regression models were offset by the number of current sexual partners for each youth.

Multivariable models were evaluated in two stages. First, interaction effects between the cumulative youth communication score and cumulative convergence measures were evaluated for each of the three SRH variables. Within each outcome variable, the item-level interaction was evaluated between every youth communication item and each convergence score for that item. Interaction effects were assessed using both p values and the change in the Bayesian Information Criterion (BIC) statistic. To assist with interpretation of the interaction effects, predicted values of the outcome variable were calculated for all potential categories pertaining to the interaction. To control for Type I errors due to multiple tests, a Bonferroni-Holm correction procedure was used in the second stage of models to determine statistical significance; continuing until the *i*^th^ ordered p value was $p\left(i\right)\ge \frac{\alpha }{K-i+1}$, at which point the value was not considered to be statistically significant [21, 22]. All analyses were conducted using STATA statistical software version 17 [23].

## Results

### Characteristics of AI Youth

Table 1 provides the characteristics of the 145 youth sampled. Seventy-two (52.4%) were male and 69 (47.6%) were female. Most (65.6%) youth were between 15 and 17 years of age. The majority (71.2%) of youth were in grades 9–11. Female youth generally had higher cumulative communication scores than males. Forty-nine (33.8%) youth reported ever having engaged in sex. Females in higher school grades were more likely to have reported engaging in sex (p < 0.05). Age at sexual debut was significantly associated with youth age among both males and females, where younger youth had a lower age at sexual debut (*p* < 0.05).

**Table 1:** Characteristics of American Indian youth (n = 145)

	All males	All females	Cumulative communication score, males	Cumulative communication score, females	Males who have ever engaged in sex	Females who have ever engaged in sex	Age at sexual debut, males^a^	Age at sexual debut, females^a^	Number of protected acts of anal or vaginal sex, males	Number of protected acts of anal or vaginal sex, females
	N = 72	N = 69	N = 67	N = 63	N = 23	N = 26	N = 23	N = 26	N = 72	N = 69
Youth characteristic	N (%)	N (%)	Mean (SD)	Mean (SD)	N (%)	N (%)	Mean (SD)	Mean (SD)	Mean (SD)	Mean (SD)
Age (years)										
≤ 14	20 (27.8%)	20 (29.0%)	6.6 (4.3)	9.3 (5.5)	3 (13.0%)	3 (11.5%)	12.7*^, b^ (1.5)	13.0*^, c^ (1.0)	0.1 (0.2)	0.0 (0.0)
15	13 (18.1%)	14 (20.3%)	9.7 (5.8)	12.2 (6.3)	4 (17.4%)	4 (15.4%)	14.5 (0.6)	14.0 (0.8)	0.6 (1.7)	0.2 (0.8)
16	21 (29.2%)	19 (27.5%)	7.1 (7.6)	10.8 (7.3)	10 (43.5%)	9 (34.6%)	15.1 (1.5)	14.7 (1.1)	0.3 (0.7)	0.0 (0.0)
17	15 (20.8%)	13 (18.8%)	6.6 (7.1)	16.4 (6.7)	4 (17.4%)	8 (30.8%)	15.7 (0.6)	15.9 (0.9)	0.1 (0.3)	0.5 (1.5)
≥ 18	3 (4.2%)	3 (4.4%)	8.7 (4.9)	13.7 (5.9)	2 (8.7%)	2 (7.7%)	13.5 (0.7)	15.5 (0.7)	1.3 (2.3)	0.3 (0.6)
School site										
Brockton	12 (16.7%)	12 (17.4%)	7.4 (7.4)	11.5 (8.0)	6 (26.1%)	6 (23.1%)	15.0 (1.0)	15.2 (1.3)	0.9 (2.0)	0.1 (0.3)
Culbertson	11 (15.3%)	2 (2.9%)	5.2 (7.1)	18.0 (0.0)	4 (17.4%)	1 (3.8%)	14.3 (2.1)	-	0.0 (0.0)	0.0 (0.0)
Frazer	3 (4.2%)	11 (15.9%)	6.0 (6.1)	9.8 (7.0)	2 (8.7%)	4 (15.3%)	14.0 (1.4)	14.8 (1.0)	0.7 (1.2)	0.0 (0.0)
Poplar	11 (15.3%)	14 (20.3%)	8.4 (5.0)	8.7 (4.3)	4 (17.4%)	3 (11.5%)	13.5 (2.1)	14.7 (0.6)	0.0 (0.0)	0.4 (1.3)
Wolf Point	35 (48.6%)	30 (43.5%)	7.9 (6.2)	13.8 (6.5)	7 (30.4%)	12 (46.1%)	15.2 (1.0)	14.6 (1.3)	0.2 (0.6)	0.2 (0.7)
Grade level										
8th	8 (11.1%)	11 (15.9%)	8.4 (3.4)	8.8 (5.5)	2 (8.7%)	1*^, d^ (3.8%)	12.0 (1.4)	13.0 (0.0)	0.0 (0.0)	0.0 (0.0)
9th	20 (27.8%)	17 (24.6%)	6.4 (5.1)	10.7 (5.6)	3 (13.0%)	5 (19.2%)	14.7 (1.2)	14.2 (1.5)	0.1 (0.2)	0.0 (0.0)
10th	15 (20.8%)	18 (26.1%)	8.9 (7.0)	11.0 (7.8)	6 (26.1%)	4 (15.3%)	15.0 (0.7)	14.3 (1.3)	0.7 (1.6)	0.2 (0.8)
11th	18 (25.0%)	16 (23.2%)	6.9 (7.7)	14.1 (5.6)	8 (34.8%)	10 (38.5%)	15.0 (1.7)	15.3 (1.4)	0.3 (0.8)	0.3 (1.3)
12th	11 (15.3%)	7 (10.1%)	6.7 (6.6)	16.2 (8.1)	4 (17.4%)	6 (23.1%)	14.5 (1.3)	15.4 (0.5)	0.4 (1.3)	0.4 (0.8)

*SD* Standard deviation

^*^p < .05

^a^Only among sexually active students

^b^*P* value derived from a one-way analysis of variance test conducted to test difference in means of age at sexual debut for males in different age groups

^c^*P* value derived from a one-way analysis of variance test conducted to test difference in means of age at sexual debut for females in different age groups

^d^*P* value derived from a Pearson’s chi-square test conducted to test difference in number of sexually active females between grade levels

### Characteristics of AI Youth According to Caregiver Characteristics

Table 2 provides the characteristics of the 113 caregivers sampled. Most (71.7%) were less than 50 years old, and most (87.6%) were female. Most caregivers (81.4%) had a 2-year or associate degree or less education. Communication scores for female youth varied significantly across school site reported by the caregiver, with higher communication scores occurring for Culbertson and Wolf Point (*p* < 0.05). Female youth with female caregivers had a higher age at sexual debut than female youth with male caregivers (*p* < 0.01).

**Table 2:** Characteristics of American Indian youth relative to caregiver characteristics (n = 145)

	All males	All females	Cumulative communication score, males	Cumulative communication score, females	Males who have ever engaged in sex	Females who have ever engaged in sex	Age at sexual debut, males^a^	Age at sexual debut, females^a^	Number of protected acts of anal or vaginal sex, males	Number of protected acts of anal or vaginal sex, females
Caregiver characteristic	N = 51	N = 60	N = 49	N = 54	N = 18	N = 21	N = 18	N = 21	N = 51	N = 60
	N (%)	N (%)	Mean (SD)	Mean (SD)	N (%)	N (%)	Mean (SD)	Mean (SD)	Mean (SD)	Mean (SD)
Age (years)										
< 40	15 (29.4%)	24 (40.0%)	8.3 (6.2)	12.1 (6.4)	5 (27.8%)	11 (52.4%)	14.3 (1.0)	14.6 (1.1)	0.1 (0.4)	0.4 (1.2)
40–49	21 (41.2%)	21 (35.0%)	8.4 (7.4)	11.2 (7.7)	6 (33.3%)	7 (33.3%)	15.0 (1.0)	14.6 (1.5)	0.4 (1.1)	0.0 (0.0)
50–59	8 (15.7%)	7 (11.7%)	4.9 (5.1)	14.2 (6.4)	5 (27.8%)	2 (9.5%)	14.5 (2.1)	15.0 (0.0)	0.0 (0.0)	0.0 (0.0)
≥ 60	7 (13.7%)	8 (13.3%)	3.9 (2.7)	11.0 (6.6)	2 (11.1%)	1 (4.8%)	14.0 (1.4)	16.5 (0.7)	0.0 (0.0)	0.0 (0.0)
Gender										
Male	6 (11.8%)	8 (13.3%)	6.7 (5.4)	8.9 (4.7)	1 (5.6%)	3 (14.3%)	16.0 (0.0)	13.6**^,d^ (1.1)	0.2 (0.4)	0.0 (0.0)
Female	45 (88.2%)	52 (88.7%)	7.2 (6.6)	12.4 (7.0)	17 (94.4%)	18 (85.7%)	14.5 (1.5)	15.1 (1.2)	0.2 (0.8)	0.2 (0.8)
School Site^b^										
Brockton	8 (15.7%)	10 (17.5%)	7.6 (7.9)	10.8*^,c^ (8.6)	4 (22.2%)	5 (25.0%)	15.0 (1.0)	15.2 (1.3)	0.5 (1.4)	0.0 (0.0)
Culbertson	8 (15.7%)	1 (1.8%)	6.5 (7.8)	18.0 (0.0)	4 (22.2%)	0 (0.0%)	14.3 (2.1)	-	0.0 (0.0)	0.0 (0.0)
Frazer	2 (3.9%)	7 (12.3%)	2.5 (0.7)	6.5 (4.7)	1 (5.6%)	2 (10.0%)	14.0 (1.4)	15.0 (1.0)	0.0 (0.0)	0.0 (0.0)
Poplar	10 (19.6%)	14 (24.6%)	7.8 (4.9)	9.2 (4.3)	3 (16.7%)	3 (15.0%)	13.5 (2.1)	14.7 (0.6)	0.0 (0.0)	0.4 (1.3)
Wolf Point	23 (45.1%)	25 (43.9%)	7.4 (6.3)	14.7 (6.2)	6 (33.3%)	10 (50.0%)	15.2 (1.0)	14.6 (1.6)	0.3 (0.7)	0.2 (0.7)
Highest level of education completed										
Did not graduate HS	2 (3.9%)	6 (10.0%)	5.0 (4.2)	15.8 (4.0)	2 (11.1%)	2 (9.5%)	16.0 (0.0)	14.0 (0.0)	0.0 (0.0)	0.0 (0.0)
GED	6 (11.8%)	13 (21.7%)	7.5 (8.5)	11.3 (8.5)	2 (11.1%)	5 (23.8%)	15.0 (1.4)	15.5 (1.3)	0.2 (0.4)	0.2 (0.8)
HS degree	19 (37.3%)	25 (41.7%)	5.2 (4.7)	10.2 (5.9)	6 (33.3%)	6 (28.6%)	13.9 (1.8)	14.3 (1.2)	0.1 (0.3)	0.1 (0.4)
2 year or AD	14 (27.5%)	7 (11.7%)	9.3 (5.6)	13.1 (6.6)	5 (27.8%)	2 (9.5%)	14.8 (0.8)	13.3 (1.2)	0.3 (1.1)	0.0 (0.0)
Bachelor’s	6 (11.8%)	7 (11.7%)	11.0 (9.9)	13.4 (7.8)	2 (11.1%)	5 (23.8%)	14.0 (0.0)	15.7 (0.6)	0.5 (1.2)	0.7 (1.9)
Master’s or higher	4 (7.8%)	2 (3.3%)	3.5 (4.4)	14.0 (11.3)	1 (5.6%)	1 (4.8%)	16.0 (0.0)	15.0 (0.0)	0.0 (0.0)	0.0 (0.0)

*SD* Standard deviation*, HS* High school*, GED* General education degree*, AD* Associate degree

^*^p < .05, **p < .01

^a^Only among sexually active students

^b^School site was reported as the school where the caregiver’s child attended

^c^*P* value derived from a one-way analysis of variance test conducted to test difference in means of communication score for females at different school sites

^d^*P* value derived from a student’s t-test to test difference in means of age at sexual debut between youth with male vs female caregivers

### Caregiver-youth Communication Patterns Regarding SRH

Table 3 contains data on self-reported communication patterns (among youth) and convergence with caregiver responses. High convergence in caregiver-youth communication was evident for the following SRH topics: *what qualities are important in choosing close friends* (94.2% convergence), *the consequences of getting pregnant or getting someone pregnant* (89.8% convergence), *menstruation* (85.5% convergence), and *how women get pregnant and have babies* (83.3% convergence). Female youth generally reported higher rates of talking about a given topic for 13 of the 24 communication topics. Appreciable variation between male and female youth responses was evident for the topics: *how girls’ bodies change physically as they grow up* (76.8% endorsed by females vs 21.4% by males), *how well birth control can prevent pregnancy* (52.2% endorsed by females vs 19.4% by males), *how to choose a method of birth control* (38.2% endorsed by females vs 8.3% by males), *menstruation* (73.1% endorsed by females vs 15.5% by males), *reasons why you should not have sex* (55.1% endorsed by females vs 25.0% by males), and *homosexuality/people being gay* (59.4% endorsed by females vs 15.3% by males).

**Table 3:** Communication between AI caregivers and youth regarding SRH items (n = 145)

	All youth (n = 145)		Males (n = 72)		Females (n = 69)	
Communication topic	Self-reported communication about topic	Convergent caregiver responses	Self-reported communication about topic	Convergent caregiver responses	Self-reported communication about topic	Convergent caregiver responses
What qualities are important in choosing close friends	86 (59.3%)	81 (94.2%)	40 (55.6%)	37 (92.5%)	44 (63.8%)	42 (95.5%)
What qualities to look for in a boyfriend, girlfriend, or life partner	70 (48.3%)	62 (88.6%)	31 (43.1%)	25 (80.7%)	38 (55.1%)	36 (94.7%)
How boys’ bodies change physically as they grow up	60 (42.0%)	39 (65.0%)	32 (45.7%)	20 (62.5%)	26 (37.7%)	17 (65.4%)
How girls’ bodies change physically as they grow up	71 (50%)	53 (74.7%)	15*** (21.4%)	8 (53.3%)	53*** (76.8%)	43 (81.1%)
How women get pregnant and have babies	78 (53.8%)	65 (83.3%)	29** (40.3%)	23 (79.3%)	47** (68.1%)	40 (85.1%)
Symptoms of sexually transmitted infections (STIs)	53 (36.6%)	39 (73.6%)	20* (27.8%)	15 (75.0%)	31* (44.9%)	22 (71.0%)
Reasons why people like to have sex	41 (28.3%)	18 (43.9%)	21 (29.2%)	8 (38.1%)	19 (27.5%)	9 (47.4%)
How well birth control can prevent pregnancy	53 (36.6%)	44 (83.0%)	14*** (19.4%)	12 (85.7%)	36*** (52.2%)	29 (80.6%)
How to choose a method of birth control	34 (23.6%)	27 (79.4%)	6*** (8.3%)	4 (66.7%)	26*** (38.2%)	21 (80.8%)
How to say no if someone wants to have sex and you don’t want to	77 (53.5%)	55 (71.4%)	28** (38.9%)	19 (67.9%)	46** (67.7%)	33 (71.7%)
How well condoms can prevent sexually transmitted infections (STIs)	65 (45.1%)	50 (76.9%)	32 (45.1%)	26 (81.3%)	31 (44.9%)	22 (71.0%)
Menstruation (having menstrual periods)	62 (43.7%)	53 (85.5%)	11*** (15.5%)	8 (72.7%)	49*** (73.1%)	43 (87.8%)
The importance of not pressuring other people to have sex	68 (47.2%)	51 (75.0%)	29 (40.9%)	22 (75.9%)	37 (53.6%)	27 (73.0%)
How you will make decisions about whether or not to have sex	67 (46.5%)	54 (80.6%)	26* (36.1%)	21 (80.8%)	38* (55.9%)	30 (79.0%)
Consequences of getting pregnant/getting someone pregnant	88 (61.1%)	79 (89.8%)	35** (49.3%)	32 (91.4%)	50** (72.5%)	44 (88.0%)
How to use a condom	42 (29.8%)	21 (50.0%)	19 (27.1%)	8 (42.1%)	21 (31.3%)	12 (57.1%)
Reasons why you should not have sex	59 (40.7%)	51 (86.4%)	18*** (25.0%)	16 (88.9%)	38*** (55.1%)	32 (84.2%)
Masturbation	18 (12.4%)	8 (44.4%)	12 (16.7%)	5 (41.7%)	6 (8.7%)	3 (50.0%)
What it feels like to have sex	21 (14.5%)	7 (33.3%)	9 (12.5%)	2 (22.2%)	12 (17.4%)	5 (41.7%)
Homosexuality/people being gay	54 (37.2%)	48 (88.9%)	11*** (15.3%)	10 (90.9%)	41*** (59.4%)	36 (87.8%)
Wet dreams [measured for boys only]	12 (8.3%)	6 (50.0%)	10* (14.1%)	6 (60.0%)	2* (2.9%)	0 (0.0%)
What to do if a partner doesn’t want to use a condom	36 (24.8%)	20 (55.6%)	14 (19.4%)	7 (50.0%)	20 (29.0%)	11 (55.0%)
How people can prevent getting sexually transmitted infections (STIs)	61 (42.1%)	50 (82.0%)	21** (29.2%)	18 (85.7%)	38** (55.1%)	30 (79.0%)
How you will know if you are in love	65 (45.5%)	45 (69.2%)	29 (40.3%)	18 (62.1%)	33 (49.3%)	24 (72.7%)

^*^p < 0.05; **p < 0.01; ***p < 0.001

*P* values derived from Pearson’s chi-square tests

### Communication and Sexual Risk Outcomes

**Fig. 1:**
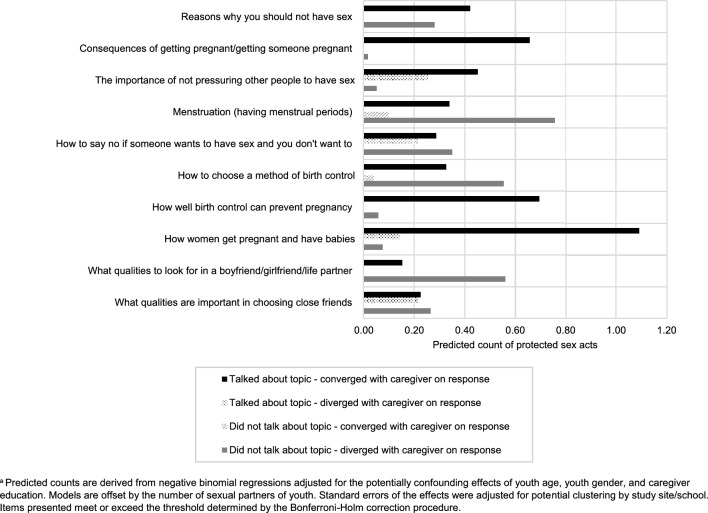
Predicted counts of protected acts of vaginal and anal sex among youth by caregiver-youth communication pattern^a^

Table 4 contains data from multivariable regression analyses involving youth sexual risk outcomes and caregiver-youth communication patterns reported in this study. Cumulative youth communication scores were significantly associated with whether the student had ever engaged in sex, with higher levels of communication being associated with an increased likelihood of ever having engaged in sex (*p* < 0.001). A significant interaction effect between youth communication and convergence with caregiver response was observed for the number of protected acts of vaginal and/or anal sex (*p* < 0.01, ∆BIC = ‒ 3.8). Figure 1 illustrates significant interaction effects for communication items in relation to condom use. Globally, caregiver communication on a topic was generally associated with higher counts of protected sex acts, regardless of youth responses. For example, for the communication item: “what qualities to look for in a boyfriend/girlfriend/life partner,” the highest counts of protected sex acts were observed among youth who reported talking about the topic and who converged with the caregiver response on that item, and among youth who reported not talking about the topic but who diverged with the caregiver response on that item (Fig. 1).

**Table 4:** Composite models for youth SRH outcomes before and after interaction terms

	Whether student has ever had sex^a^		Age at sexual debut^b,c^		Number of protected anal or vaginal sex acts^d^	
Independent variable	Model 1	Model 2	Model 3	Model 4	Model 5	Model 6
	*logit (SE)*	*logit (SE)*	*beta (SE)*	*beta (SE)*	*log of the count (SE)*	*log of the count (SE)*
Cumulative youth communication score	0.13*** (7.52)	0.10 (1.17)	‒ 0.01 (0.05)	‒ 0.26 (0.15)	0.15*** (5.70)	0.43*** (3.84)
Cumulative convergence score for caregiver and youth communication	0.01 (0.31)	‒ 0.02 (‒ 0.23)	0.03 (0.09)	‒ 0.14 (0.17)	0.04 (0.35)	0.32* (2.39)
Interaction between youth communication and convergence score		0.00 (0.48)		0.02 (0.01)		‒ 0.02** (‒ 2.84)
Student age	0.58** (3.25)	0.57*** (3.41)	0.55 (0.26)	0.44 (0.27)	0.51** (3.19)	0.55*** (4.28)
Sex of the student^e^	0.12 (0.21)	0.15 (0.28)	‒ 0.08 (0.36)	‒ 0.07 (0.27)	0.82 (1.16)	0.87 (1.23)
Highest level of education completed by caregiver^f^	‒ 0.19** (‒ 3.15)	‒ 0.19** (‒ 3.08)	‒ 0.16 (0.06)	‒ 0.27 (0.13)	‒ 0.08 (‒ 0.29)	‒ 0.12 (‒ 0.45)
Constant	‒ 10.50*** (‒ 4.10)	‒ 10.10*** (‒ 4.78)	6.13 (3.93)	10.49 (4.59)	‒ 12.87***(‒ 4.85)	‒ 17.39*** (‒ 6.38)
lnalpha					0.99 (1.48)	0.47 (0.47)
BIC	151.6	151.4	141.5	135.3	110.2	106.4
N	129	129	40	40	126	126

*BIC* Bayesian Information Criterion Statistic, *SE* Standard error

^*^p < 0.05; **p < 0.01; ***p < 0.001

^a^Estimates are logit coefficients derived from a logistic regression adjusted for the potentially confounding effects of youth age, youth gender, and caregiver education, and the standard errors of the effects were adjusted for potential clustering by study site/school

^b^Estimates are beta weights derived from an OLS regression adjusted for the potentially confounding effects of youth age, youth gender, and caregiver education, and the standard errors of the effects were adjusted for potential clustering by study site/school

^c^Analyses were only conducted among sexually active students

^d^Estimates are logarithms of the count derived from a negative binomial regression adjusted for the potentially confounding effects of youth age, youth gender, and caregiver education. Models are offset by the number of sexual partners of youth. Standard errors of the effects were adjusted for potential clustering by study site/school

^e^Reference group: female students

^f^Reference group: Did not graduate high school

## Discussion

The purpose of the current study was to examine caregiver-youth communication patterns in relation to SRH outcomes among AI youth. While more self-reported communication by youth was associated with engagement in sex, caregiver communication was largely associated with condom use among youth. Results have several implications for developing Indigenous-forward policy and future experimental studies evaluating SRH outcomes among AI youth.

First, the interaction effects detected in this current study suggest that regardless of what youth report, it is caregiver communication that was most strongly associated with condom use during sex. This finding underscores the importance of caregiver communication for youth sexual risk behavior. In RCTs conducted among non-AI parents and adolescents in the United States, several have reported on the effectiveness of interventions that focus on parental involvement and communication to impact youth SRH [24]. Current literature includes studies among youth-parent dyads with similar age groups, yet differing racial/ethnic characteristics that support the results from this study [13, 14, 25, 26]. One cluster-RCT conducted with a similar youth age group among African Americans reported a delay in sexual initiation among the younger youth and increased condom use among the older youth [13]. In another study, there was a dose effect of parent-youth sexual risk communication and adolescent outcomes including condom use skills [14]. While there are limited RCTs with AI youth that examine SRH, these existing studies support the evidence of increased parent-youth communication impacting youth sexual risk behavior and may be appropriate models of comparison to support the findings of our study. Evidence from this current study suggests that communication about SRH topics reported by a caregiver shows the strongest association with youth condom use behavior, and future experimental studies with AI youth should consider how changes in caregiver communication may impact changes in youth condom use over time.

Separately, higher levels of self-reported youth communication with caregivers about SRH were associated with an increased likelihood of ever having sex in this current study. Past studies show similar associations and suggest that parents or youth are more likely to initiate conversations as youth become more sexually active [2–4, 27–30]. After youth become sexually active, parent communication may moderate the negative effects of peer pressure on condom use [31, 32], and help youth establish effective communication patterns, more protective sexual practices, and less risky sexual behavior as youth get older and more sexually active [33–37]. Specifically, within the context of NE, the RCT from which these baseline results are derived, focusing on the potential benefit of caregiver-youth communication for improving youth SRH outcomes has deeper cultural and historical implications [17]. A component of NE emphasizes the AI cultural practice of caregivers passing down SRH knowledge to their youth through an Indigenous cosmological framework of SRH while also working to shift the content and timing of caregivers’ conversations with youth [1, 17]. Caregiver-youth communication historically emphasized traditional roles for youth in parenthood, especially regarding what is expected of youth after their coming of age. In Northern plains tribes, coming of age ceremonies were one of the seven sacred rites of White Buffalo Calf Woman and celebrated youth transition into adulthood by explaining their new roles and responsibilities as young adults and what was expected of them as young adults in the community. It is important to support the present-day practice of such traditional cultural practices within tribal communities while integrating caregiver-youth conversations into present-day needs and cultural shifts, where STIs are more prevalent, sexual relationships are more varied among youth, sexuality is acknowledged to exist on a broader spectrum, and gender roles are more mixed between males and females.

A strength and contribution of this current study is that it separately assesses communication patterns in AI caregivers and youth to evaluate how communication patterns relate to youth sexual risk behaviors. Other studies that have only evaluated parental communication in non-AI populations have failed to detect associations between unilaterally evaluated parental communication and condom use or birth control [28, 30]. Some studies in non-AI populations have found that parental communication is associated with youth sexual risk behaviors, but effects were not as pronounced as the current study. Bonafide et al. most recently found a small but significant association between parent-reported communication and less youth condomless sex in suburban African American youth (B = ‒ 0.01, *p* = 0.01), but after controlling for youth-reported communication, this association became insignificant. Furthermore, a small but significant negative association was found between youth-reported communication and youth condomless sex, but only when parent responses were congruent with youth responses [38]. While results from the current study are relatively consistent with this finding, item-level analyses of individual SRH communication topics reveal a more complex relationship between caregiver-youth response congruence and youth condom use, where this relationship changes depending on the SRH topic discussed. For example, congruent responses confirming caregiver-youth communication regarding topics including *consequences of getting pregnant/getting someone pregnant* and *how well birth control can prevent pregnancy* were congruent with Bonafide et al.’s study, where only congruent responses predicted more protected sex acts for youth. However, incongruent responses between caregivers and youth, where caregivers confirmed communication and youth did not, regarding topics including *what qualities to look for in a boyfriend/girlfriend/life partner* and *menstruation* predicted more protected sex acts in youth. Results suggest that when measuring caregiver and youth responses separately, it is helpful to examine the specific topic discussed. Furthermore, considering the impact that colonial violence has specifically had on caregiver-youth dynamics, as well as the more significant role that caregivers have in guiding youth through their coming of age, results from the current study may vary due to population-specific and cultural differences from those reported in other studies [1].

### Limitations

This study has several limitations. First, this was a cross-sectional study from baseline data derived from NE, and changes in communication or behavior could not be evaluated. Second, results cannot be generalized to other AI communities. AI youth living on the Fort Peck Reservation may be missing one or both biological parents, making comparative analyses between caregivers in the current study and parents in other studies a potentially challenging endeavor, especially considering whether the person responding to the current study survey was a biological parent, aunt, uncle, friend, or other caregiver. Furthermore, youth who may transfer between caregivers or have multiple caregivers responding to the same survey should be monitored throughout the course of the intervention. Third, the caregiver population in this study may not be representative of all caregivers on the reservation, given the low rate of caregiver participation. Additionally, non-participating caregivers were not surveyed, which prevents further investigation into the low rate of caregiver participation. Fourth, specific demographic caregiver data was not collected for this study, which prevented any delineation between parents, grandparents, or non-biological caregivers. Fifth, lower 12th grade class numbers across school sites may be suggestive of school dropout, which could have biased the outcomes of this study. AI students across Montana continue to experience high dropout rates when compared to non-AI students [39]. For AI communities, dropout is a symptom of many factors, including historical trauma from the boarding school era, transportation challenges, mistrust with the current school system, high student mobility, and lack of administrative support for AI students, and pregnancy is not generally listed as a common factor to consider when looking into AI dropout rates [40, 41]. On the Fort Peck Reservation, early childbearing is perceived as a less negative consequence than in non-AI communities, because youth understand that their family will support them in the case that they become pregnant. Kinship networks and family support at Fort Peck lead to more positive experiences of early childbearing for AI youth [42]. Although the current study did not explore specific reasons for dropout in the schools at Fort Peck, future qualitative and quantitative endline analyses will investigate changes in caregiver-youth communication patterns and how they relate to various primary, secondary, and tertiary SRH outcomes for AI youth. Sixth, the caregiver survey asked caregivers if they had talked to their youth about a specific topic, but did not allow room for expansion. For example, if caregivers selected “yes” to *reasons for why you should not have sex*, they could not further expand on what sort of reasons they discussed with their youth for why they should not have sex. This warrants further investigation because topics discussed between caregivers and their youth could vary between families and may reveal new complexities in caregiver communication patterns and youth SRH behavior that this study could not uncover. Finally, youth communication, youth sexual behavior, and caregiver communication were based on self-report. In many AI communities, relationships and community setting influence how participants communicate, and participant response accuracy may depend on the level of trust they have for who is present during survey collection. Having AI community partners present for survey collection and the use of computer-assisted self-interview were methods used to mitigate these biases, but other unobserved factors could affect reporting and thus the relationships reported.

## New Contribution to the Literature

The purpose of this study was to evaluate how caregiver-youth communication patterns were related to sexual risk behaviors among AI youth. Results suggest that caregiver communication with youth is associated with more condom use. This study fills a gap in the existing literature by reporting on relationships between communication about SRH, assessed separately in AI caregivers and youth, and youth sexual risk behaviors. Findings emphasize the importance of involving AI caregivers in SRH interventions, especially considering the role that the relationship between AI caregivers and youth has in developing youth participants’ understanding of their coming of age and related SRH topics. While concurrently addressing the negative effect that the legacy of colonial violence has left on AI populations in the US, findings will help develop culturally centered interventions that improve SRH outcomes among AI youth while reinforcing AI cultural values and family structure. Findings from this study inform future intervention research to evaluate how changes in AI caregiver communication—especially those that reintegrate traditional cultural practices back into present day caregiver-youth conversations—potentially impact youth SRH over time.
